# Case Report: First in Human Online Adaptive MR Guided SBRT of Peritoneal Carcinomatosis Nodules: A New Therapeutic Approach for the Oligo-Metastatic Patient

**DOI:** 10.3389/fonc.2020.601739

**Published:** 2020-12-15

**Authors:** Luca Boldrini, Angela Romano, Lorenzo Placidi, Gian Carlo Mattiucci, Giuditta Chiloiro, Davide Cusumano, Veronica Pollutri, Marco Valerio Antonelli, Luca Indovina, Maria Antonietta Gambacorta, Vincenzo Valentini

**Affiliations:** Dipartimento di Diagnostica per Immagini, Radioterapia Oncologica ed Ematologia, Fondazione Policlinico Universitario “Agostino Gemelli” IRCCS, Rome, Italy

**Keywords:** online adaptive radiation therapy, oligometastatic disease, stereotactic body radiation therapy, magnetic resonance guided radiation therapy, peritoneal metastases, colorectal cancer

## Abstract

Peritoneal carcinosis (PC) is characterized by poor prognosis. PC is currently treated as a locoregional disease and the possibility to perform very precise treatments such as stereotactic body radiation therapy (SBRT) has opened up new therapeutic perspectives. More recently, the introduction of Magnetic Resonance-guided Radiation Therapy (MRgRT) allowed online adaptation (OA) of treatment plan to optimize daily dose distribution based on patient’s anatomy. The aim of this study is the evaluation of the effectiveness of SBRT OA workflow in an oligometastatic patient affected by PC. We report the clinical case of a patient affected by PC originating from colon cancer, previously treated with chemotherapy and surgery, addressed to OA SBRT treatment on a single chemoresistant PC nodule, delivered with a 0.35 T MR Linac. Treatment was delivered using gating approach in deep inspiration breath hold condition in order to reduce intrafraction variability. Prescription dose was 35 Gy in 5 fractions. The PTV V95% of the original plan was 96.6%, while the predicted values for the following fractions were 11.9, 56.4, 0, 0, and 61%. Similarly, the small bowel V19.5 Gy of the original plan was 4.63 cc, while the predicted values for the following fractions were 3.7, 8.6, 10.7, 1.96, 3.7 cc. Thanks to the OA approach, the re-optimized PTV V95% coverage improved to 96.1, 89.0, 85.5, 94.5, and 94%; while the small bowel V19.5 Gy to 3.36; 3.28; 1.84; 2.62; 2.6 cc respectively. After the end of RT, the patient was addressed to follow-up, and the re-evaluation ^18^F-FDG PET-CT was performed after 10 months from irradiation showed complete response. No acute or late toxicities were recorded. MRgRT with OA approach in PC patients is technically and clinically feasible with clean toxicity result. Online adaptive SBRT for oligometastases opens up new therapeutic scenarios in the management of this category of patients.

## Introduction

Peritoneal carcinomatosis (PC) is the second most common cause of death in patients affected by colorectal cancer (CRC) and can be diagnosed at the time of diagnosis (synchronous PC) or during follow-up (methacronous PC) (1, 2).

Metachronous PC is detected in 4–19% of patients (3) and isolated peritoneal metastases are observed in 10% of them. Survival rates in this group of patients remain poor, with a median survival of only 12 months despite systemic therapies (1–4).

PC has historically been considered a widespread disease presentation, with peculiar molecular mechanisms for the different primary diseases.

Novel therapeutic approaches emerged during the last two decades for patients presenting PC, such as cytoreductive surgery (CRS) in combination with hyperthermic intraperitoneal chemotherapy (HIPEC) (5). The multimodal approach with systemic therapy and local treatment should currently be proposed to patients presenting systemic stable disease, in which local ablative approaches may result in disease control (6).

Many studies report survival benefits for CRS and HIPEC (5, 7) and these techniques can be therefore considered the standard of treatment in PC patients presenting localized disease (6, 8). Elias et al. described a 2-year and 5-year overall survival rates of 81 and 51% in patients undergoing CRS and HIPEC, respectively, *versus* 65 and 13% in patients receiving chemotherapy (5).

However, reliable clinical data regarding oncological outcomes are still lacking and procedural mortality and morbidity rates, ranging from 0.9 to 5.8% and 12 to 52%, respectively (9), impose the need of careful patient selection, as underlined also by Sugarbaker et al. (10).

Although PC management has changed in recent years, as it is now considered a locoregional disease rather than a metastatic disease presentation, the role of radiotherapy is still limited, and other treatment modalities (*i.e.* CRS and HIPEC) are preferred.

Ablative localized treatments represent the basic principle of the current management of oligometastatic disease presentation, a status defined by the presence of an intermediate disease state, between localized and advanced metastatic. Guckenberger et al. recently presented a dynamic model of oligometastatic disease. According to this model, oligometastatic disease can occur at different times in patient’s clinical history, intersecting with systemic and local therapies (6, 11).

In this framework, the therapeutic decision should be shared in a multidisciplinary tumor board, defining a virtuous succession of systemic and local ablative treatments.

As for typical oligometastatic diseases, the careful patient selection is mandatory also for PC. The extension of peritoneal seeding, expressed by the peritoneal carcinomatosis index (PCI) and the histology of primary tumor [*e.g.* mucinous adenocarcinoma and signet ring cell carcinomas (SC) are characterized by worse prognosis] represent important prognostic factors that should be evaluated when addressing the patient to local therapies (8, 12, 13).

Radiotherapy has historically been used in the treatment of PC in both adjuvant or palliative settings for ovarian, endometrial, and gastrointestinal cancers, having whole abdomen as target volume (14). The first experiences date back to the 80s and 90s and are based on obsolete delivery technologies (^60^Cobalt or orthovoltage units) that did not allow doses sufficient for disease control (15–18).

The most commonly used doses were 30 Gy in 1–1.5 Gy/fraction, with dose escalation possibility with a 16–20 Gy boost on the primary tumor site in compliant patients (14).

The introduction of intensity modulated radiotherapy (IMRT) created new opportunities for PC treatment with RT, thanks to the more conformal dose distribution to the target and the reduction of unnecessary irradiation of the organs at risk (OARs) (14).

Radiation therapy delivery technology has dramatically progressed to the possibility of performing image-guided radiotherapy (IGRT) and stereotactic body radiotherapy (SBRT), allowing more precise radiation treatment delivery and dose escalation. More recent developments have led to the possibility to take advantage of on-board Magnetic Resonance Imaging (MRI) combined with Linear Accelerator (MRI-Linac Systems), especially with small target hardly visible with standard techniques, as often occurs in SBRT of abdominal targets (19, 20).

Magnetic Resonance guided Radiation Therapy (MRgRT) allows superior soft-tissue contrast and online cine-MRI guidance to be combined with gating techniques. This promotes cutting edge motion management strategies which enable the daily performance of plan re-optimization prior to the delivery of each fraction (Online Adaptive Radiation Therapy) (21, 22).

## Case Description

### Oncological History

The case of a 77-year-old woman with no family history of cancer and a prior medical history only significant for autoimmune hepatitis is here presented.

Due to the onset of constipation, she underwent colonoscopy which showed the presence of a malignant sigmoid lesion in July 2018. The finding was later confirmed by ^18^F-FDG PET-CT scan in September 2018.

The patient then underwent laparoscopic left hemicolectomy with nodal dissection (October 2018), with the pathological examination showing a moderately/poorly differentiated (G2/G3) stage II (pT3pN0) sigmoid adenocarcinoma with negative resection margin (pR0) and no vascular invasion (V0), microsatellite instability (MSI) not detected, RAS and BRAF wild type.

The patient was sent to clinical-instrumental follow-up with no indication for adjuvant treatments.

The first ^18^F-FDG PET-CT follow-up scan acquired on January 2019 showed increased FDG accumulation in two nodules at the level of the mesentery tissue and widespread pseudo nodular peritoneal thickening (left side) compatible with PC implants.

Chemotherapy was then started with FOLFOX (Oxaliplatin 85 mg/mq intravenous infusion—5-fluorouracil 2,400 mg/mq intravenous infusion over 46 h—Folinic Acid 200 mg/mq intravenous infusion) plus Panitumumab (6 mg/kg) scheme, with a total of seven foreseen cycles.

The last two cycles have been administered with a 25% dose reduction due to the onset of G1 diarrhea, G2 oral neuropathy, G2 hand-foot syndrome, and G1 stomatitis, according to CTACE v 4.0.

After seven cycles of chemotherapy, maintenance therapy with 5-FU and Panitumumab was prescribed.

The ^18^F-FDG PET-CT scan of May 2019 showed only a focal uptake in correspondence of a nodular formation of 12 × 8 mm with a standardized uptake value (SUV) of 20.7 in the context of the mesenteric adipose tissue in the left hemi abdomen in close proximity to small bowel loops.

The serum levels of CA 125, CEA and CA 19-9 were in the normal range.

**Figure 1:**
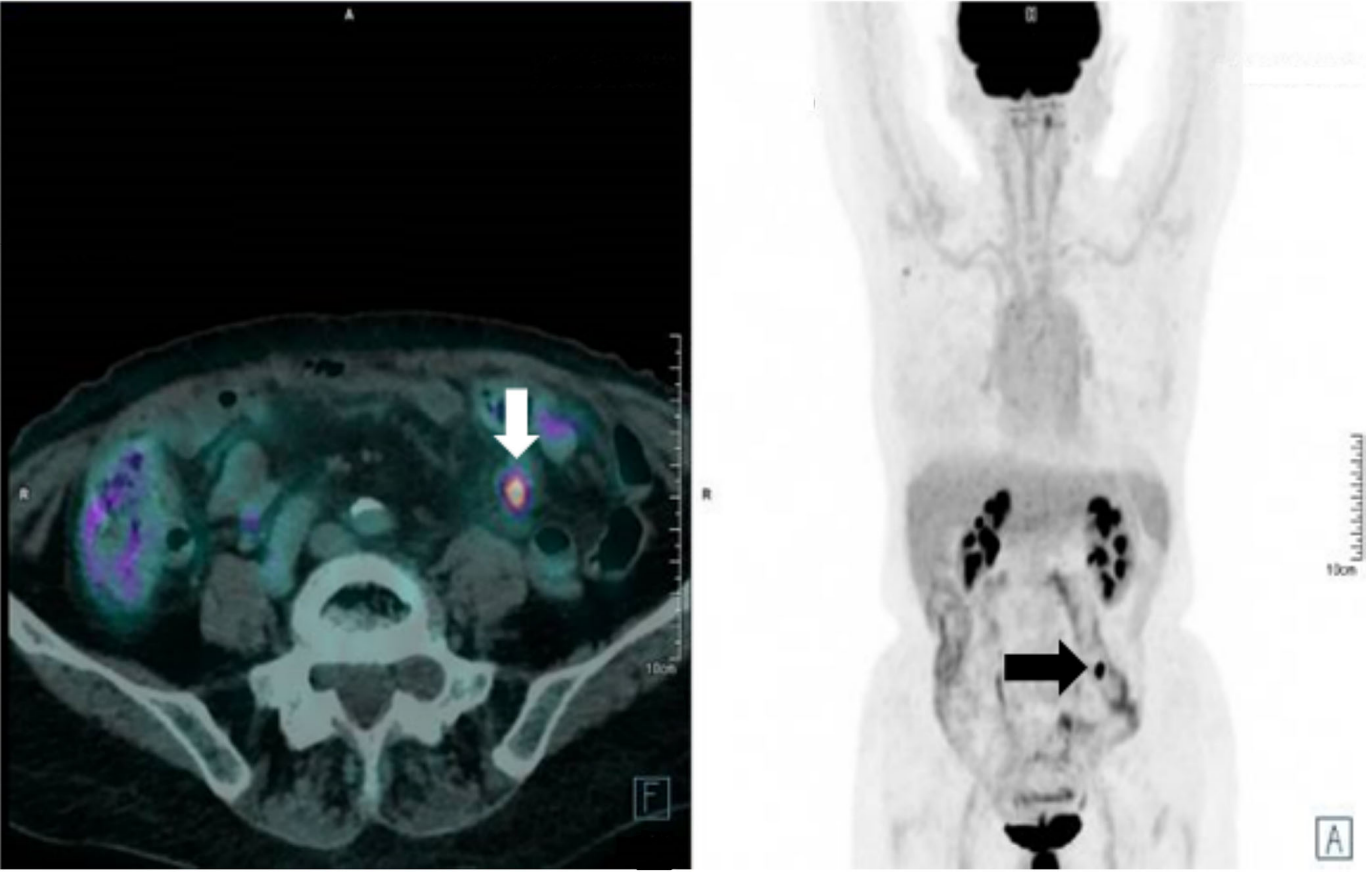
Staging PET-CT used for target definition. The lesion is clearly visible in the hypogastric-left iliac fossa area (see arrows).

The case was discussed in the institutional multidisciplinary tumor board that excluded surgery and addressed the patient to SBRT on the single PET positive peritoneal carcinomatosis nodule (see **Figure 1**).

The patient was consequently evaluated for MRI compliance [MASTER score value 1 (23)] and addressed to 0.35 T 6 MV hybrid MR-Linac (MRIdian, ViewRay Inc., USA) with fully online adaptive approach.

### Radiotherapy Simulation

The patient was positioned in supine position using the FluxBoard immobilization device (FluxBoard™, MacroMedics, the Netherlands) in the most reproducible and comfortable arrangement. Dedicated MRI coils were positioned under and over the abdomen of the patient. A simulation MRI was acquired according to our internal protocol.

A first 25-s true fast imaging (TRUFI) MR scan in free breathing (FB) was used to confirm patient’s correct positioning. A following 25-s deep inspiration breath hold (DIBH) sequence was then acquired (FOV of 54 × 47 × 43 cm^3^).

Target motion was verified with a 60-s cine-MRI on a sagittal plane (the only one currently allowed by the MRIdian system) passing through the GTV center of mass. The observed maximum amplitude was 4 mm in both cranio-caudal (CC) and antero-posterior (AP) directions.

The Gross Tumor Volume (GTV) (1.52 × 1.57 × 1 cm) included the persistent uptake in the mesenteric adipose tissue, as shown by ^18^F-FDG PET-CT scan. No margins for the clinical target volume (CTV) were applied (GTV = CTV). The surrounding OARs were contoured according to our institutional guidelines (RTOG).

Planning Target Volumes (PTVs) were obtained by adding a 3 mm isotropic margin to the GTV and ITV.

Considering such expansion, the PTV originated from GTV showed an 8.9 cc volume in DIBH, while the PTV obtained from ITV would have reached a 13.2 cc volume.

The patient then underwent a standard non-contrast enhanced planning CT in DIBH conditions, within 30 min since the MRI simulation on a helical CT scanner (GE HiSpeed DX/i Spiral, Boston, MA, USA, 1.5 mm slice thickness).

This planning CT scan was then co-registered with the simulation MR using deformable registration algorithms in order to obtain electron density data required for dose calculation.

### Radiotherapy Planning

A SBRT treatment plan with seven fields and 60 segments was calculated.

Dose calculation was carried out with a full Monte Carlo algorithm (MRIdian, ViewRay Inc., USA), with a statistical uncertainty of 1% using a calculation grid of 0.2 cm × 0.2 cm × 0.2 cm (24–26). The mean time required to obtain an initial dose distribution that solved the cost function was 1.6 ± 0.5 min (24).

Planning quality was assessed using target coverage objectives and OAR constraints as recommended by the AAPM Task Group 101 report (27). Small bowel loops hard constraints were set as Dmax < 35 Gy and V19.5% < 5 cc.

A fully online adaptive MRgRT workflow was chosen considering the site of target lesion, which was surrounded by numerous small bowel loops. Plan was calculated in DIBH in order to mitigate the breathing related movements on the CC axis observed during simulation imaging acquisition and to reduce target volume extension (28).

A 25 s TRUFI sequence positioning MRI has been acquired prior to each delivery fraction to ensure patient’s correct positioning. The original contours were then automatically propagated on the positioning MRI of the day and manually edited within a distance of 3 cm from PTV to ensure therapy volume consistency, according to the SMART approach (29).

The fluence of the original or last delivered plan was recalculated on the daily anatomy prior to each fraction, to evaluate the predicted dose distribution and the relative DVH parameters. In case of non-compliance with the set constraints, the dose distribution was re-optimized online.

Intra-fraction target motion was managed through an automatic gating approach triggered by target volume displacement (gating boundary = PTV; target out % = 5%) and verified using the online cine-MRI (four frames per second, sagittal view).

A total dose of 35 Gy at 7 Gy per fraction was prescribed to PTV, according to ICRU 83 guidelines.

**Figure 2:**
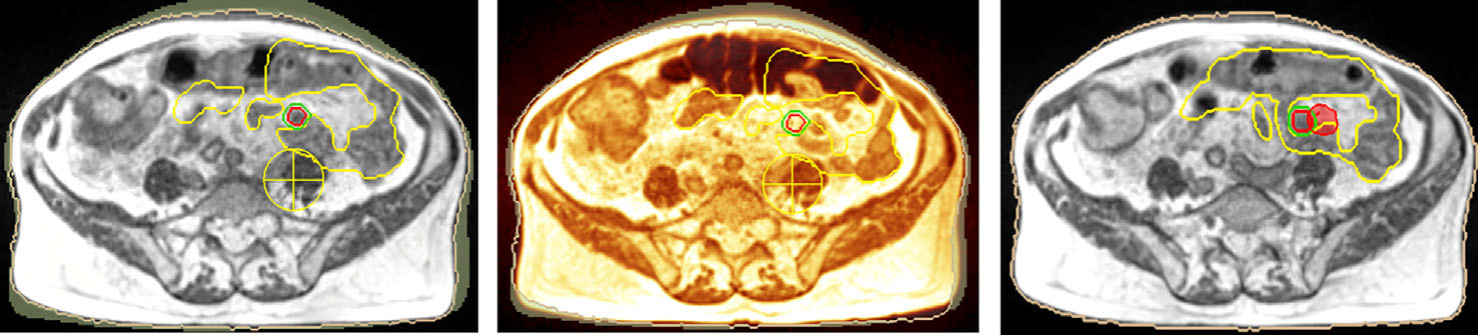
Simulation imaging (left), first fraction positioning imaging (middle) (red: GTV, green: gating boundary, yellow: small bowel loops). Target lesion is not visible and dose distribution is inconsistent (right, 95% isodose level in red colorwash).

All the scheduled fractions needed to be re-optimized due to significant dose distribution inconsistencies both for OAR constraints respectively and target coverage (see **Figure 2**).

The mean dose reduction in small bowel Dmax achieved through online adaptive replanning was 3.1 Gy (range 0.2–8.7 Gy), and the mean reduction in small bowel V19.5 Gy was 3 cc (range 0.39–8.86) comparing to the predicted dose if treated with the original plan without adaptation.

As for target coverage, the mean PTV V95% increase was 65.9% (range 32.6–94.5%) (see **Figure 2**, right).

The dosimetric differences between predicted and re-optimized plans are reported in **Table 1** for each delivered fraction.

**Table 1:** Dosimetric differences between predicted and re-optimized plans.

Volume	Original plan	Plan	Fraction 1	Fraction 2	Fraction 3	Fraction 4	Fraction 5
**PTV V95%**	96.6%	Predicted	11.9%	56.4%	0%	0%	61%
		Re-opt	96.1%	89.0%	85.5%	94.5%	94%
**Small bowel Dmax (Gy)**	34.2	Predicted	33.8	36.5	38.1	33.9	36.5
		Re-opt	33.6	33.7	29.4	34.2	32.2
**Small bowel V19.5 Gy (cc)**	4.63	Predicted	3.75	8.6	10.7	1.96	3.7
		Re-opt	3.36	3.28	1.84	2.62	2.6

Original beam on time (as calculated by the TPS) was 3.62 minutes (min) while the predicted delivery time was 5.50 min.

**Figure 3:**
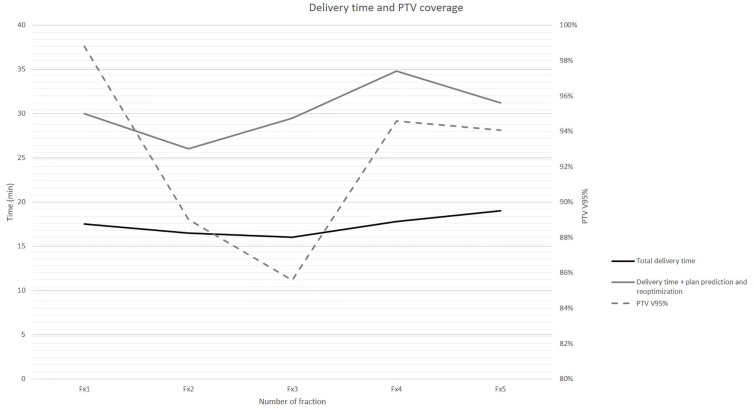
Beam on time and total delivery time (primary axis, in min) for each following adapted treatment fraction reported against achieved PTV coverage (secondary axis, in PTV V95%).

Beam on time and delivery time for each following adapted fraction are summarized in **Figure 3**.

The alignment and positioning image acquisition, contouring, plan prediction, and plan reoptimization and total delivery times resulted: 7.7 min (7–8.2); 8.2 min (7–9); 12.9 min (9.5–17); 17.3 min (16–19), respectively.

Patient’s on couch total time was 46.2 min (range 40–52).

The patient underwent the treatment without interruptions. No toxicity was observed during irradiation or at follow-up assessments, according to. CTCAE 4.0 scale (30).

After SBRT, the patient was addressed to follow-up, consisting of a complete examination and recording of the clinical history, laboratory tests and restaging imaging, with no other active oncological treatments.

A first re-evaluation ^18^F-FDG PET-CT acquired on September 2019 (2 months after irradiation) described a slight reduction in both lesion’s dimension and standardized uptake value (SUV) (10 × 7 and 7.5 mm respectively) while a second ^18^F-FDG PET-CT acquired on May 2020 (10 months after irradiation) showed complete response with no other active disease sites.

The following last ^18^F-FDG PET-CT of September 2020 (14 months after irradiation) confirmed the observed local complete response in the absence of other disease sites.

## Discussion

This case describes an innovative multimodal treatment approach, for a patient affected by PC.

PC is a complex condition, characterized by specific biological behaviour, dependent on the underlying primary disease, for which the most appropriate therapeutic approach is still not clear.

The recent PRODIGE trial compared CRS and HIPEC *versus* CRS alone and showed no differences in terms of 5-year overall survival between the two arms, while the 1-year relapse free survival rate was 46.1% in the non-HIPEC arm *vs* 59% in the HIPEC arm, underlining the importance of surgery for this patients category (8, 31).

The presented patient developed PC three months after radical surgery of the primary tumor and was subsequently addressed to systemic therapy, obtaining only a partial response on PC site [induced oligoprogressive disease, as for Guckenberger et al. (11)].

The first therapeutic choice would have been therefore to perform CRS (with or without HIPEC) since the patient presented a chemo-responsive disease, with PCI <12 and without SC histology (6, 32).

As our patient was found to be unfit for surgery, the multidisciplinary tumor board shared the decision to address her patient to SBRT on the persisting ^18^F-FDG-PET positive PC nodule.

This decision has been made possible thanks to the use of online adaptive MRgRT.

This hybrid solution allowed delivery of the prescribed SBRT treatment that could not have been performed using traditional RT techniques due to the significant mobility of the target, its challenging anatomical identification, and its position relative to the radiosensitive surrounding OARs.

The main limit of this report relies in the description of an uncommon clinical condition treated with RT delivery technology that is currently available only in very few institutions all over the world. Nevertheless, it shows future perspectives on the RT management of localized PC thanks to the use of such innovative technology.

The proposed approach achieved optimal results in terms of disease control (16 months DFS at the time writing), which are comparable to those obtained with much more invasive PC treatments (*e.g.* CRS combined with HIPEC or CRS alone), and this advantage is even more significant considering that no toxicity has been reported, in view of the high toxicity rate that burdens surgical approaches (31).

These findings suggest that online MRgRT of PC nodules is feasible and has promising results in the oligometastatic setting that supports the design of further research trials on the specific topic.

## Data Availability Statement

The original contributions presented in the study are included in the article, further inquiries can be directed to the corresponding author.

## Ethics Statement

Ethical review and approval was not required for the study on human participants in accordance with the local legislation and institutional requirements. The patients/participants provided their written informed consent to participate in this study.

## Author Contributions

LB, AR, and GC were responsible for the radiotherapy treatment workflow and delivery. GM, VV, and MG dealt with the management of multidisciplinary tumor board pathology and indication for treatment. DC, LP, and LI managed the radiotherapy treatment plan processing and data analysis. VP and MA managed the radiotherapy treatment in terms of simulation, patient positioning, and delivery. All authors contributed to the article and approved the submitted version.

## Conflict of Interest

LB and DC have active research agreements with ViewRay Inc and received speaker honoraria for scientific presentations. VV has received departmental research grants from Varian Medical Systems, ViewRay Inc., Elekta, Merck-Serono, and Roche.

The remaining authors declare that the research was conducted in the absence of any commercial or financial relationships that could be construed as a potential conflict of interest.
